# Discussion

**Published:** 1904-02

**Authors:** 


### DISCUSSION.

Dr. Walker: I am interested in porcelain work and would like to know how to get out the matrices without twisting them.

Dr. Byers: The matric is removed in the ordinary way with an exploring point, or any finep oint, that will go under the edge and tease it out. I do not find very much difficulty in taking it out, if there is no undercut. If there is, you will have a hard time. The cavity must be on obsolutely true lines, and then it is a very easy matter to pick it out.

Dr. Barker: Perhaps a little hint that has helped me out of many a tight place may be worth while mentioning. I do not use platinum foil; I use gold foil for matrices. In deep cavities it is very apt to bend. I pack the matrix full of cotton, tight, with a cotton ball rammed in, and then drop melted wax on the cotton. You can put a hooked instrument into the cotton or the wax and pull it out. Even if you have wax and no cotton, it will hold the matrix in position and keep it from being moved. It may puzzle you to get the wax and cotton out of the matrix. Do not try it. Invest it in asbestos and burn it out—melt it out. There may be a little ash, which you can blow out with a puff of the breath; but there is nothing left that will cause trouble.

Dr. Ottolengui: I think the idea of making this porcelain piece first and pushing it through the matrix is not at all a bad one, and rather ingenious. I will say a word about the removal of the matrix with something filled in it. We must be careful in filling a matrix with anything like wax, to use something that does not contract. We put the material in with the idea of having the matrix retain its shape. If you use wax, you will be apt to draw it out of shape. I have heard recommended this month, a mixture of wax and paraffine, so as to have a combination that would soften very readily. It is to be used in a mushy consistency and pressed in almost cold. The method of getting rid of it was a little different from Dr. Barker's way. The matrix is placed in the furnace without being invested; a little piece of spunk being placed on top. With a very little heat, the material melts and is absorbed by the spunk, and there is a fairly clean matrix. I have tried the method of burning and have not been successful always in getting everything out. There may be something in the cotton or wax that is not entirely destroyed by combustion, and when it is heated up, it may adhere to the surface of the gold. That was why I abandoned it. I believe Dr. Walker will find if he continues to practice porcelain inlay work that he will have less and less need for wax or anything else. It is largely a matter of shaping the cavity and getting space. I always think the recommendation to have the teeth well wedged apart is a good one. It is also a good idea to have the gum forced away.

